# Targeted HPTLC Profile, Quantification of Flavonoids and Phenolic Acids, and Antimicrobial Activity of *Dodonaea angustifolia* (L.f.) Leaves and Flowers

**DOI:** 10.3390/molecules28062870

**Published:** 2023-03-22

**Authors:** Fekade Beshah Tessema, Yilma Hunde Gonfa, Tilahun Belayneh Asfaw, Mesfin Getachew Tadesse, Tigist Getachew Tadesse, Archana Bachheti, Mohammed O. Alshaharni, Pankaj Kumar, Vinod Kumar, Ivan Širić, Sami Abou Fayssal, Kundan Kumar Chaubey, Rakesh Kumar Bachheti

**Affiliations:** 1 Department of Industrial Chemistry, Addis Ababa Science and Technology University, Addis Ababa P.O. Box 16417, Ethiopia; 2 Centre of Excellence in Biotechnology and Bioprocess, Addis Ababa Science and Technology University, Addis Ababa P.O. Box 16417, Ethiopia; 3 Department of Chemistry, Faculty of Natural and Computational Science, Woldia University, Woldia P.O. Box. 400, Ethiopia; 4 Department of Chemistry, Faculty of Natural and Computational Science, Ambo University, Ambo P.O. Box 19, Ethiopia; 5 Department of Chemistry, College of Natural and Computational Science, Gondar University, Gondar P.O. Box 196, Ethiopia; 6 Bio and Emerging Technology Institute, Health Biotechnology Directorate, Addis Ababa P.O. Box 5954, Ethiopia; 7 Department of Environment Science, Graphic Era (Deemed to be University), Dehradun 248002, India; 8 Department of Biology, College of Science, King Khalid University, Abha 61321, Saudi Arabia; 9 Agro-Ecology and Pollution Research Laboratory, Department of Zoology and Environmental Science, Gurukula Kangri (Deemed to Be University), Haridwar 249404, India; 10 University of Zagreb, Faculty of Agriculture, Svetosimunska 25, 10000 Zagreb, Croatia; 11 Department of Agronomy, Faculty of Agronomy, University of Forestry, 10 Kliment Ohridski Blvd, 1797 Sofia, Bulgaria; 12 Department of Plant Production, Faculty of Agriculture, Lebanese University, Beirut 1302, Lebanon; 13 Division of Research and Innovation, School of Applied and Life Sciences, Uttaranchal University, Arcadia Grant, P.O. Chandanwari, Prem Nagar, Dehradun 248007, India

**Keywords:** antimicrobial activities, *Dodonaea angustifolia*, flavonoids, medicinal plants, phenolic contents

## Abstract

In East Africa, *Dodonaea angustifolia* (L.f.) is a well-known medicinal herb. Its leaf is primarily studied in light of its ethnobotanical use. In terms of phytochemistry and biological activity, its flower is not studied. In a prior study, our team looked into phytochemical screening, antioxidant activity, and total phenolic levels. This study aims to compare the profiles and biological activities of the leaf and flower samples of *D. angustifolia* and to present therapeutic alternatives. The leaf and flower sample powders were extracted with methanol using ultrasound-assisted extraction (UAE). HPTLC profile was obtained using CAMAG—HPTLC equipped with VisionCATS software. Antimicrobial agar well diffusion assay and minimum inhibition concentration (MIC) were determined. The leaf and flower extracts of *D. angustifolia* showed antibacterial activity with a MIC value of 20 µg/mL against *Enterococcus faecalis* and *Listeria monocytogenes*. Similarly, 40 µg/mL was found to be effective against *Aspergillus flavus*. *D. angustifolia* flower is a rich source of flavonoids and phenolic acids. Because of its antibacterial properties and profile, which are almost the same, the flower is emerging as a viable option for medicinal alternatives.

## 1. Introduction

*Dodonaea angustifolia* (L.f.; Syn: *Dodonaea viscosa*) (Sapindaceae) is a variable shrub or tree that grows 2 to 8 m tall. It has branchlets that are rusty red and resinous, and dark grey, fissured, peeling bark. The leaf is narrowly elliptic, with a tapering apex and base and an entire border, with alternately or spirally arranged leaves. The petiole of the leaves, which are up to 10 mm long, is narrowly elliptic and bright green above. Young twigs and leaves are sticky and resinous. Leaves are simple lanceolate, light green, with toothless margins, round or pointy leaf tips; 5–10 cm long; 5–8 mm wide; the leaves secrete a sticky fluid which makes them appear to always shine. Fruits are roundish, greenish-red capsules that are about 2 cm in diameter. They have two or three small, inconspicuous, papery-winged flowers that are pale green, with greenish-yellow sepals but no petals, and brown stamens [1]. Sand olives are hermaphrodites in nature; the wind distributes the seeds. The plant has biophysical limits: 0–2 800 m above sea level, 450 mm on average each year of rainfall, and rocky or deficient soil types. The plant is native to Australia, Ethiopia, Kenya, New Zealand, Oman, South Africa, and Tanzania [1]. *D. angustifolia* is known by different names in different places: Ethiopian (Karkare, Agew), Kitkitta (Amh, Gur), Termien (Geez), Ettecca (Oro), Intanca (Sid), Tahses (Tre, Tya, Den, Som) [2,3,4]; English (sticky hopbush, sand olive); Hindi (pulivavila); Swahili (mkaa-pwani) [1].

In Latin America, China, Africa, and India, *D. angustifolia* aerial parts are used as a traditional medicine to treat fevers, swellings, and colds [5,6]. Roundworms and trachoma are both treated with powdered leaf juice [7]. Traditional medical systems use this plant’s parts for contraceptives [8]. Its antimalarial effect is also significant in different parts of Ethiopia [9,10]. To treat rheumatism, the stems are fumigants [7].

The leaf extracts in ethanol, methanol, ethyl acetate, chloroform, and aqueous form are effective against fungus [11,12]. The leaf essential oil and extracts have significantly inhibited *Staphylococcus aureus*, *Micrococcus luteus*, and *Escherichia coli* [7,13]. The root’s antidiarrheal properties have been demonstrated by the root’s alcohol and aqueous extract, which greatly reduced diarrhea in mice and resulted in lighter feces [14]. The leaves showed analgesic [7], antidiabetic [6,15,16,17], anti-inflammatory [18,19], antioxidant [20,21], antipyretic [22], antiviral [23,24], hypotensive [7], and wound healing [25] properties. There is evidence that *D. angustifolia* has the potential to be an allelopathic plant, as extracts of this plant can inhibit the germination and growth of other plants [26,27,28]. On the other hand, *D. viscosa* residues had a definite favorable impact on the characteristics of growth, yield, yield components, chlorophylls, carotenes, protein, oil, and element content [29].

Phytochemical analysis of *D. angustifolia* (including its synonym) leaves revealed the presence of chemicals from several different classes of secondary plant metabolites, including flavonoids, terpenoids, saponins, tannins, cardiac glycosides, steroids, etc. [30]. From the leaves of *D. angustifolia*, more than 40 chemicals including flavonoids and terpenoids have been reported [28]. Antioxidant activity is the basis for many processes that result in the prevention of the majority of diseases. Today’s high-performance thin-layer chromatography (HPTLC) technique is incorporated into the pharmacopeial analysis. Usually, HPLC, HPTLC, and UV spectrophotometric procedures are employed to estimate flavonoids and phenolic acids for their therapeutic impact on a qualitative and quantitative level. WHO introduced the use of chromatography for standardizing plant products, and it is now recognized as a tactic for identifying and assessing the quality of plant medicines (WHO/PHARM 92.559). The results could be handled in automated and manual ways, such as with peak profiles from an image, densitogram, or an image (chromatogram) [31]. Due to its benefits of low operating costs, high sample throughput, simplicity, speed, minimal sample cleanup requirements, reproducibility, accuracy, and dependability, HPTLC is increasingly being used as a standard analytical technique for both qualitative and quantitative determinations [32].

Studies on the effects of various flavonoids and phenolics in illnesses like pneumonia, cancer, and amoebic dysentery are now being conducted. Because of their inherent structural diversity for health and dietary significance, quick and accurate assessment of phenolic acids and flavonoids is crucial [33]. Our previous investigation reported that both the leaves and flower methanol extracts of *D. angustifolia* exhibit significant antioxidant activity [34]. Phenolic acids play a crucial role in controlling plant development and disease resistance. In addition to acting as antibacterial agents and signal molecules for beneficial microbes for plants, flavonoids also serve as colors to draw in insect pollinators [35]. Yellow flavones and flavanols are particularly significant in this regard. Flavonoids shield plant tissue from UV exposure because of their powerful UV radiation absorption. Pharmacological studies have shown that flavonoids have antiviral, anti-allergic, anticancer, antibacterial, antifungal, and antithrombotic properties [33,34,36]. They affect blood arteries, specifically flavanones and catechins, which boost capillary resistance. They exhibit an anti-inflammatory action that is dependent on their structural makeup [37].

As one can see from the reports [5,6,7,8,9,10], mainly the leaves of *D. angustifolia* and its synonym are solely investigated, whereas the flowers and other parts are ignored. No HPTLC profile was reported for *D. angustifolia* leaves or flowers, or antimicrobial activity for the flowers. In this study, *D. angustifolia* leaf and flower HPTLC profile and antibacterial properties will be compared. Antimicrobial activity and the results of the Prediction of Activity Spectra for Substances (PASS) and the HPTLC profile and the HPLC profile from our earlier investigation will also be compared.

## 2. Results

### 2.1. HPTLC Analysis

From a prior investigation on the flower of *D. angustifolia* [34], it was revealed that almost similar phytochemical screening results show both the leaf and flower are rich in phenolic acids and flavonoids. Furthermore, we have compared both samples’ antioxidant activity and total phenolic and flavonoid contents. These results are summarized in the table below (Table 1). Both the leaf and flower samples show valuable phenolic and flavonoid content with slight variations.

**Figure 1:**
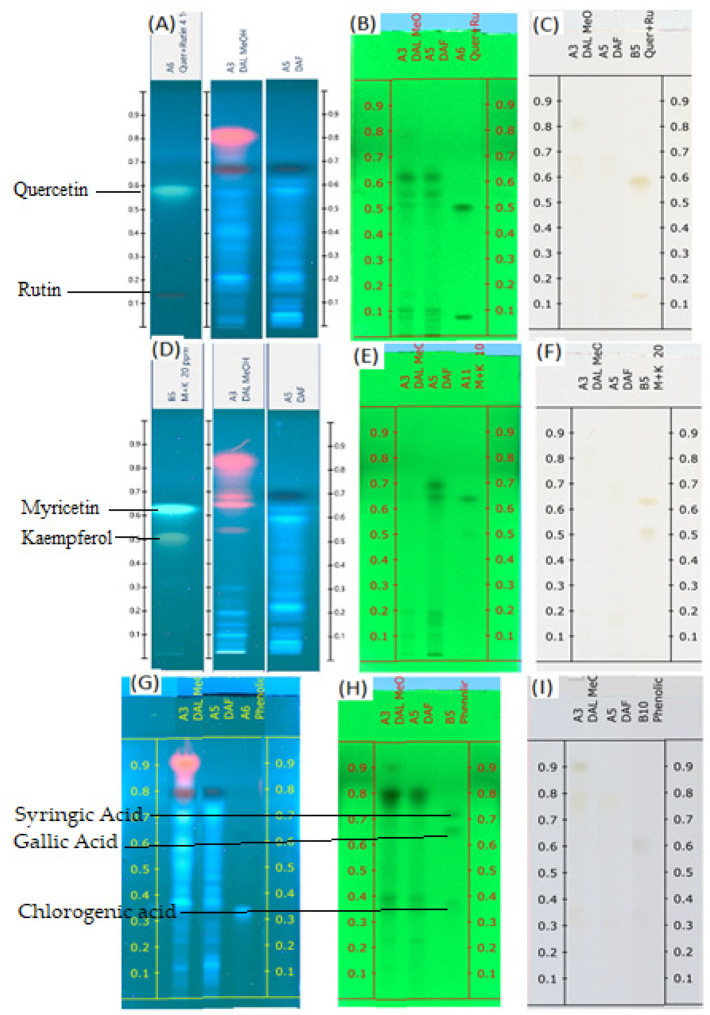
TLC profiles of DAL and DAF (**A**) Flavonoids (quercetin and rutin) at 366 nm; (**B**) Flavonoids (quercetin and rutin) at 254 nm; (**C**) Flavonoids (quercetin and rutin) at visible light; (**D**) Flavonoids (myricetin and kaempferol) at 366 nm; (**E**) Flavonoids (myricetin and kaempferol) at 254 nm; (**F**) Flavonoids (myricetin and kaempferol) at visible light; (**G**) Phenolic acids at 366 nm; (**H**) Phenolic acids at 254 nm; (**I**) phenolic acids at visible light.

The mobile phase consisting of toluene: ethyl acetate: formic acid and methanol (20:12:8:4, volume percent) for flavonoids and toluene: ethyl acetate: formic acid and methanol (10:15:10:5, volume percent) for phenolic acid showed acceptable resolution and separation of the components of the sample. Figure 1A–I displays the TLC profiles of both samples under visual conditions at 245 nm and 366 nm. The flavonoid standards were divided into quercetin and rutin in one group and myricetin and kaempferol in the other group. This grouping is based on the Rf of individual flavonoids.

**Figure 2:**
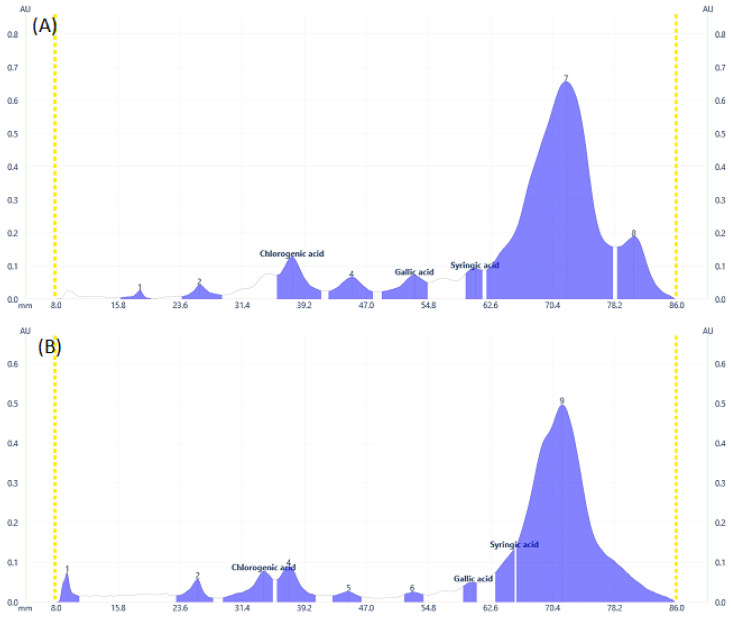
Phenolic acids profile at 273 nm for **(A**) DAL, (**B**) DAF samples.

**Figure 3:**
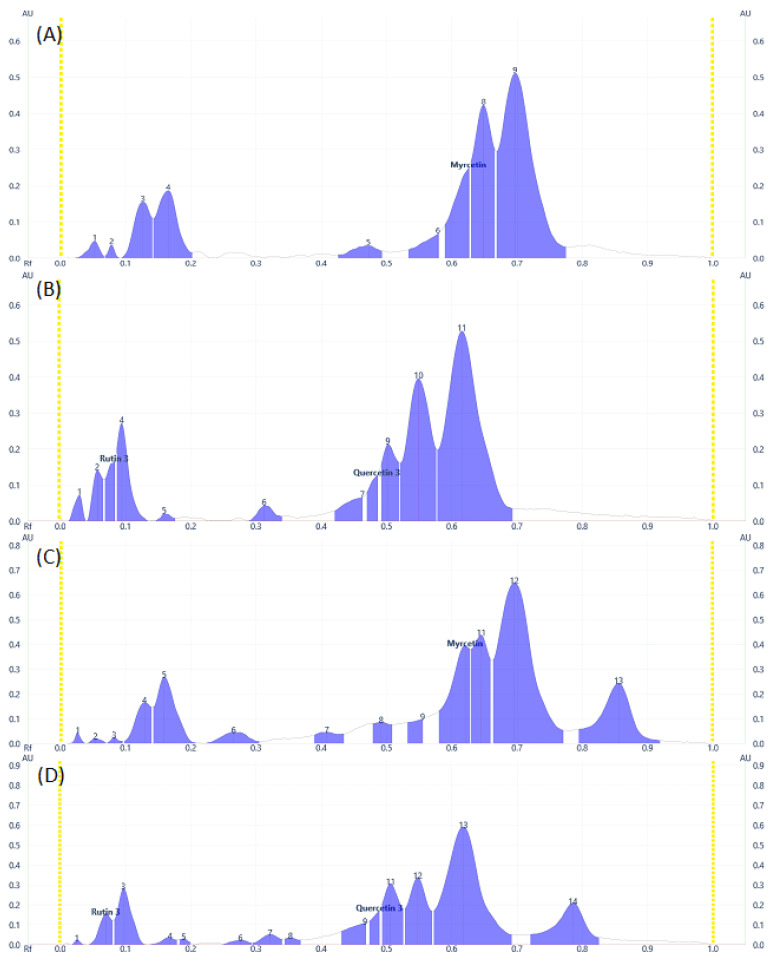
Flavonoids Profile of samples at 366 nm for (**A**) and (**B**) DAF, (**C**) and (**D**) DAL samples.

Some flavonoid and phenolic acids were even visible under the visual range as shown in Figure 1C,F,I at higher concentrations. Lower concentrations of some analytes may contribute to the disappearance of spots. The number of components detected for both samples is almost comparable. *D. angustifolia* flower contained three out of nine components identified as phenolic acids (Figure 2A,B) and four flavonoids out of eleven components (Figure 3A–D).

From the methanol extract of *D. angustifolia* leaves, out of eight components, three phenolic acids were identified (Figure 2) and 14 components were detected, among which three flavonoids were identified (Figure 3).

The validation data for the HPTLC analysis is presented in Table 2 and summarized as follows. Linearity for the phenolic acids was recorded in the ranges of 0.9912–0.9977 and for flavonoids 0.9862–0.9965. In both cases, R^2^ was close to unity. LOD values ranged from 0.0264 to 0.1317 (µg/100 mL) and LOQ values ranged from 0.0801 to 0.2182 (µg/100 mL). For two of the flavonoids (quercetin and rutin), recovery tests were managed based on the smaller number of available standards.

The quantitative results for both samples are close enough and ranged from 24 to 188 mg/100 g, shown in Table 3. The difference in content can be supported with further analysis in the future. In most cases, the contents were a bit higher for the leaf sample than for the flower. The lowest content is for kaempferol and gallic acid.

The biological activity of the flavonoids and phenolic acids is taken into consideration in this investigation and was also predicted in a prior study. Those activities concerning antimicrobial activities were as shown in Table 4. The probability of activity for all components is shown to be between 0.700–0.300.

### 2.2. Antimicrobial Activities

For both the leaf and flower extracts inhibition was observed for two gram-positive bacterial strains and one fungal strain. The inhibition zone ranges from 9.0 to 24 mm (Table 5). The maximum zone of inhibition is shown for the flower extract of *D. angustifolia*. Three gram-negative bacterial strains and one gram-positive bacterial strain were not found to be sensitive for both extracts up to a concentration of 1000 mg/mL. (See Supplementary Materials).

MIC and MBC determination were managed for those active strains for both extracts. MIC values ranged from 20 to 40 μg/mL whereas MBC ranged from 40 to 80 μg/mL. The result summary of MIC and MBC using the microdilution method is presented in the table below (Table 6).

## 3. Discussion

Studies on the phytochemical investigation and biological activities of the flower of *D. angustifolia* are lacking, while there is no report on the HPTLC profile of the leaves and the flower extracts of this plant. Therefore, the current study addresses the research gap in the HPTLC profile of the leaves and the flower extracts of *D. angustifolia*. Despite having a poorer resolution than the HPLC method, HPTLC is frequently employed in investigating pharmaceuticals, plants, foods, and environmental and clinical samples [38]. It is a prominent analytical tool for fingerprint analysis and for quantifying marker molecules in herbal medications due to its simplicity, accuracy, and adaptability for high-throughput screening [38]. Optimization of the mobile phase was done using various solvents including those suggested by scholars for flavonoids and phenolic acids. The optimization mainly focused on the developing solvent ratio. The mobile phase used by Chewchinda and Kongkiataiboon [38] with slight modification consisting of toluene: ethyl acetate: formic acid: methanol at different ratios for flavonoids and phenolic acids showed acceptable resolution and separation of the components of the samples.

The validation parameters for HPTLC analysis were found to be in an acceptable range for the purpose indicated [39]. The specificity of the standards in the target extract was confirmed by overlay UV spectra between the reference standard and sample. The limits of detection (LOD) and quantification (LOQ) were small enough, which indicates the adequate sensitivity of the method (Table 3). Results from recovery studies were within acceptable limits (96.77–99.74%), indicating that the accuracy of the method was good [39]. Therefore, this method can be considered fairly sensitive concerning these compounds [40].

In our previous study, using HPLC analysis, we identified and quantified the three phenolic acids, including chlorogenic acid, gallic acid, and syringic acid, in methanol extract of *D. angustifolia* flower extract, which is in close agreement with the current HPTLC analysis result [34]. Similar to the HPLC data presented in our earlier work, the HPTLC profiles (Figure 2 and Figure 3) and peak tables (supplementary file, Tables S1 and S2) provide information regarding the presence of flavonoids and phenolic acids. From the preliminary phytochemical screening test result [34], the leaves and flowers of *D. angustifolia* were nearly similar in terms of phytochemical groups (phenolic acids and flavonoids) considered in this investigation. The screening investigation revealed that the main components of the *D. angustifolia* leaf and flower extract included flavonoids and phenolic acids [41]. The hydroxyl groups in the structures of the flavonoids and phenolic acids can lead to better antioxidant action. As shown in Table 1, the antioxidant capacity, TPC, and TFC values of *D. angustifolia* leaves and flowers are slightly different from one another.

Flavonoids can be found in most plant parts, with photosynthetic plant cells serving as the primary source of color in blooming plants [42]. Compared to the leaves, which contain more chlorophyll and associated components, the complexity of the matrix in flowers may be lower. The quantitative data (Table 4) likewise follow the same pattern as the flower HPLC results from our earlier analysis [34]. Flavonoids in food, like quercetin and myricetin, protect against oxidative stress and aging [43]. The flavonoids under investigation in our study are considered dietary flavonoids and have many health benefits, including their use as supplements [43,44,45]. Similarly, phenolic acids are also considered important dietary components of the human diet [46,47,48]. The flower of *D. angustifolia* especially, as a rich source of these groups of phytochemicals, can be further studied as a possible candidate for a pack of nutraceuticals.

A chromatographic fingerprint created by the HPTLC on plant extracts represents the chemically unique or pharmacologically active elements present in the plants [49]. The chromatographic fingerprints of specific flavonoids and phenolic compounds were examined using the HPTLC method. The amounts of these polyphenolic groups were quantified in the methanol extracts of *D. angustifolia* leaves and flowers. Comparison of HPTLC fingerprints of methanolic extracts showed similarity, despite differences in chlorophyll’s appearance close to the front position [31]. A similar trend was observed in our case as shown in Figure 1A,D,G. The HPTLC fingerprints (Figure 1) and profile (Figure 2 and Figure 3) showed the presence of comparable components apart from some non-polar components in the leaf extract.

The variations of these bioactive concentrations are not consistent, as also reported by Alam et al. [50]; they are expected to vary due to harsh conditions and organ-specific biosynthesis. This method is sensitive, feasible, and cost-effective, and can be applied for robust analyses of quercetin-, rutin-, myricetin-, kaempferol-, chlorogenic acid-, syringic acid-, and gallic acid-containing products. This is the first time that four flavonoids and three phenolic acids have been separated simultaneously utilizing their respective groups’ best mobile phases. The flower is found to be rich in separable polar components. Following this targeted profile, a further phytochemical investigation may bring other groups of bioactive components with useful biological activities.

Biological activities (antibacterial and antifungal activities) predicted by PASS showed Pa values in the range of 0.300–0.700 (Table 5). Following this prediction both the leaves and flowers of *D. angustifolia* can be considered potential pharmacological agents [51]. The leaves are used therapeutically while the flowers are not. The antioxidant activity of the flavonoids and phenolic acids was predicted to be higher and the DPPH assay also confirmed the same trend for both the leaf and flower samples. According to Mizzi et al. [52], phenolic compounds have several physiological qualities that are known to be advantageous, including antibacterial, antioxidant, and preservation capabilities. The body’s cardiovascular, anticarcinogenic, gastroprotective, anti-inflammatory, and antibacterial functions are all dictated by antioxidant capabilities [53]. Due to their antioxidant capabilities, rutin and quercetin, for instance, show gastroprotective effects [54]. From the prediction data of antimicrobial activity (Table 4), myricetin and rutin were found to be more potent, and such recommendations were not reported so far. Jan et al. [49] reported antifungal and bacterial properties of kaempferol and quercetin. Further investigation demands the specific role of the components, especially for anti-aspergillosis activity, using molecular docking studies and in vitro assays.

PASS prediction indicated moderate antimicrobial and antifungal activities for some components considered in the current investigation. This is supported by the antimicrobial efficacy test with sensitivity for just two bacterial strains and one fungus species (Table 6). Specifically, rutin and chlorogenic acid are more active for both antimicrobial and antifungal activities. From the antimicrobial efficacy test, two of the bacterial strains (*E. faeccals and L. monocytogenes*) and *Aspergillus flavus* were found to be sensitive to both extracts. From the zone of inhibition data, the antifungal activity for the flower was found to be 24 mm, whereas for the leaves it was 16 mm. From this, we might infer that the flower will have a more notable impact on aspergillosis prevention. *E. coli* and the other two gram-negative bacteria were not found to be sensitive to antimicrobial efficacy tests up to 1000 mg/mL concentration of the extracts. *E. coli* is already known to be multi-resistant to drugs [55] and was also resistant to both plant extracts in the present study. Two gram-positive bacterial strains of enterobacteria known as foodborne pathogens were shown to be sensitive to both extracts. *E. faecalis* lives in the gastrointestinal tract and is responsible for nosocomial infections like urinary tract infections [56]. Similarly, *L. monocytogenes* causes listeriosis in animals including humans [57]. From the observed biological activities and MIC values of less than 100 μg/mL (Table 6), the flower extract can be considered an alternative for these therapeutic effects. *Aspergillus flavus* is an opportunistic fungus that affects both humans and animals, especially those with impaired immune systems. Next to *A. fumigatus*, *A. flavus* infection is now the second-most common cause of human aspergillosis. *A. flavus* produces aflatoxins, which are toxic to human health and are the cause of invasive aspergillosis in both animals and humans.

*Aspergillus flavus* produces a variety of secondary metabolites, including aflatoxins, cyclopiazonic acid, aflatrem, aflavinin, kojic acid, aspergillic acid, neoaspergillic acid, β-nitropropionic acid, and paspalinine [58]. Aflatoxin has been classified as a human liver carcinogen by the International Agency for Research on Cancer due to its risk to human health and livestock productivity [59]. *A. flavus* has been found in a variety of foods due to its extensive distribution, including dried vine berries, sour lime, cocoa beans, smoked dry meat items, cured ham, dried salted fish, and spices. [58]. Aflatoxin poisoning of agricultural products results in significant and ongoing economic damage throughout the world. Having comparable sensitivity for the two gram-positive bacteria there is a significant difference in terms of sensitivity towards *A. flavus*. The lowest minimum inhibition concentration of leaf and flower extracts of *D. angustifolia* is 20 μg/mL for *Enterococcus faecalis* and *Listeria monocytogenes*. Similarly, Lulekal et al. [60] revealed that concentrations ranging from 512 to 4 μg/mL of leaf extracts of *D. angustifolia* did not show MIC against test bacteria. The leaf and flower extracts of *D. angustifolia* showed sensitivity against *A. flavus* with a maximum inhibition zone of 16 and 24 mm. The leaf and flower extracts of *D. viscosa* showed sensitivity only against *A. flavus* with a maximum inhibition zone of 11 mm [61]. The flower extract showed a more significant effect and can be considered for use against aspergillosis when compared to the leaf extract.

## 4. Materials and Methods

### 4.1. Chemicals, Reagents, Materials, and Equipment

Distilled water was purified by MQ (18.2) with a 21 °C water purification system (Purelab flex 4, Elga, Woodridge, IL, USA). All the extraction solvents were AR grade, while for HPTLC analysis, HPLC-grade solvents and reagents were used. Standards for phenolic acids include syringic acid, chlorogenic acid, and gallic acid; flavonoid standards include myricetin, quercetin, rutin, and kaempferol; they were purchased from Sigma (>99.9%, Sigma-China, Kaohsiung City, Taiwan, China). Intelligent ultrasonic processor—(SJIA-950W, Ningbo Sjia Lab Equipment Co., Ltd, Ningbo, China) TLC Densitometer CAMAG (Muttenz, Switzerland) equipped with Automatic TLC Sampler—ATS 4, TLC scanner 4, TLC Visulaizer 2, Automatic Developing Chamber—ADC2, VisonCATS server lab data system, version 2.3.16286.1 software and precoated silica gel 60 F254 aluminum plates (20 × 10 cm, 100 µm thickness; Merck, Darmstadt, Germany) were used in the study.

For the antimicrobial study barium chloride (Lab Kemical, Ambala, India), brain heart infusion broth (BHI), nutrient broth (Accumix, Jaipur, India), mannitol salt agar (SRL, Mumbai, India), Mueller Hinton agar (HIMEDIA, Thane, India), nutrient agar (Accumix, Jaipur, India), sodium chloride (MERCK, Darmstadt, Germany), distilled water, 70% alcohol, DMSO (Sigma-Aldrich, Bangalore, India), MacConkey agar (Accumix, Jaipur, India), Sabouraud Dextrose broth (SDB) (CDH, New Delhi, India), Sabouraud Dextrose Agar (SDA) (CDH, New Delhi, India), violet red glucose bile agar (SRL, India), sulfuric acid (LOBA Chemie, India), Simon’s citrate agar (SRL, Mumbai, India), triple sugar iron agar (HIMEDIA, India), motility agar (Aplpa Chem, Ambala, India) and common laboratory components like Biosafety hood cabinet (BIOBASE, Jinan, China), incubator (BIOBASE, Jinan, China), autoclave (LABO- HUB China), vortex mixer, microscope, micropipettes (Supertek Scientific, Saha, India), plates, pipette tips, Eppendorf tubes, falcon tubes, and glass tubes were used.

### 4.2. Plant Material Collection and Pre-Treatment

The leaves and flower parts of *D. angustifolia* were collected from Addis Ababa Science and Technology University Campus (VRP5+3W6, 88852° N 38. 8098° E, Elevation: 2840 m). The samples were washed with tap water after collection and then with distilled water to remove dirt and other debris. After that, the samples were cut into smaller pieces (<45 μm) and spread out at room temperature (23 ± 3 °C) onto clean polyethylene plastic sheets. A stainless-steel sample grinder (700 g Electric grinding Mill, USHA Int., Gurgaon, India) was used to grind the air-dried samples. The plant was identified by Mr. Melaku Wondafrash, and a herbarium sample was deposited at the National Herbarium at the College of Science, Addis Ababa University, Ethiopia (Voucher number: FB-001/11).

### 4.3. Ultrasonic-Assisted Extraction (UAE)

Air-dried and powdered samples of *D. angustifolia* (L.f.) leaf and flower methanol extracts were obtained from a UAE (Probe Φ6) sonicator. About 5 g of each leaf and flower powder sample were extracted in 25 mL methanol. In this process, the temperature, duration, and power rate for sonication were set to 35 °C, 15 min, and 50%, respectively. In addition, each aliquot of the extracts was subjected to two rounds of consecutive sonication before the extracts were centrifuged using pro-analytical at 600 (10× *g*) for 20 min. Finally, the supernatant was then filtered using Whatman no. 1 filter paper, adjusted to a volume of 50 mL, and stored in the refrigerator in an amber vial for subsequent examination.

### 4.4. HPTLC Analysis

#### 4.4.1. Preparation of Standard Solutions

For the analysis of both phenolic acids and flavonoids, calibration curves using external standards were prepared. For all standards, stock solutions (400 mg/L for flavonoids and syringic acid, 1000 mg/L for gallic and chlorogenic acids) were prepared and used for serial dilutions to make the ranges of working calibration standards [32]. More briefly, a stock solution of standards was prepared by dissolving 2 mg of precisely weighed standards in methanol and bringing the volume up to 5 mL with methanol for flavonoids and syringic acid. Stock solutions of gallic and chlorogenic acids containing 1000 mg/L were also prepared by dissolving 100 mg in 100 mL of methanol and kept at 4 °C.

#### 4.4.2. Chromatography Plate Layout

The stationary phase used was Merck, HPTLC plates silica gel 60 F 254, with a plate format: 200.0 × 100.0 mm Application: Position Y: 8.0 mm, length: 4.0 mm, width: 0.0 mm Track: First position X: 12.0 mm, distance: 8.4 mm, Solvent front position: 85.0 mm.

#### 4.4.3. Chromatographic Conditions

The chromatographic stationary phase was a precoated silica gel 60 F254 HPTLC aluminum plate (20 × 10 cm, 0.1 mm thick 5–6 µm particle size; Merck, Darmstadt, Germany). The mobile phase composition was toluene: ethyl acetate: formic acid: and methanol (20:12:4:8, volume ratio). 20 µL and 2 µL of the standard solutions and samples, respectively, were spotted using ATS 4 autosampler fitted with a 100 µm Hamilton syringe. The plates were developed to a distance of 85 mm using the optimized mobile phase in CAMAG Automatic Developing Chamber (ADC 2) pre-saturated with the mobile phase. Saturation time was 20 min with 25 mL of mobile phase in a pad. Activation was done by MgCl_2_ (33% RH) for 10 min. The developed plates were dried for 5 min, with pre-drying, and preconditioned for 5 min. For densitometry measurements, spectra recording, and data processing, a CAMAG TLC Scanner 4 equipped with VisionCATS planar chromatography manager software was utilized to scan the dry plate. At a scan rate of 20 mm/s, the absorption/remission measuring mode was employed. Using deuterium and tungsten lamps, the standards and samples’ absorption spectra were recorded from 254 to 450 nm. The HPTLC analysis was performed at 25–26 °C and 44–46% humidity.

#### 4.4.4. Calibration Curve for Standards

The standard solutions (50 to 1600 ng/band) were applied in triplicates on the HPTLC plate. The plate was developed and scanned according to the chromatographic conditions indicated above. The peak areas were recorded. Calibration curves were prepared by plotting the peak area versus the concentration of the standards applied.

#### 4.4.5. HPTLC Analysis of Plant Extract

Samples of methanolic extract of *D. angustifolia* leaves and flowers were filtered through a 0.45 µm filter. HPTLC analysis was performed under the conditions optimized based on the solvent system recommended by Chewchinda et al. [38] with modifications for the reference compound. The number of analytes in the plant part extracts was quantified using a calibration curve plotted with the respective standards.

#### 4.4.6. Validation of the Method

HPTLC method was validated according to the ICH guideline [62]. Linearity was investigated with standard stock solution aliquots ranging from 200 to 1000 ng/band. Peak area versus concentration data was plotted to create the calibration curves. The least-square linear regression analysis was applied to the peak areas. The following equations (Equations (1) and (2)) were used to establish the limit of detection (LOD) and limit of quantification (LOQ):

(1) $$\mathrm{LOD}=3.3\times \frac{\mathrm{Standard}\;\mathrm{Deviation}\;\mathrm{of}\;\mathrm{the}\;y-\mathrm{intercept}}{\mathrm{Slope}\;\mathrm{of}\;\mathrm{the}\;\mathrm{calibration}\;\mathrm{curve}}$$

(2) $$\mathrm{LOQ}=10\times \frac{\mathrm{Standard}\;\mathrm{Deviation}\;\mathrm{of}\;\mathrm{the}\;y-\mathrm{intercept}}{\mathrm{Slope}\;\mathrm{of}\;\mathrm{the}\;\mathrm{calibration}\;\mathrm{curve}}$$

Relative standard deviation (RSD) was used to express the results. By figuring out the component’s peak purity, the specificity of the approach was confirmed. Recovery studies were conducted at three levels in triplicate to evaluate the method’s accuracy. An amount of standard that is known was added for recovery experiments [32].

### 4.5. Antimicrobial and Antifungal Activities

#### 4.5.1. Antimicrobial Efficacy Test

The antimicrobial efficacy study for the plant extract was carried out in the microbiology lab of the Bio and Emerging Technology Institute (BETin), Addis Ababa, Ethiopia.

#### 4.5.2. Bacterial Strains and Local Strains of Fungal Species

The antimicrobial activities of the plant extracts were tested against gram-negative bacteria (*Escherichia coli* (ATCC25972), *Klebsiella pneumonia* (ATTC70063), *Proteus mirabilis* (ATCC35659), and *Pseudomonas aeruginosa* (ATCC27853)) and gram-positive bacteria (*Listeria monocytogenes* (ATCC19115), *Staphylococcus aureus* (ATTC25923) and *Enterococcus faecalis* (ATCC79112)). These bacterial strains were obtained from Ethiopian Public Health Institute, Addis Ababa, Ethiopia. The bacterial strains were maintained in nutrient agar and Mueller-Hinton agar sub-cultured weekly to make sure that fresh cultures were used each time for inoculum preparation. *Aspergillus flavus* and *Fusarium* spp. fungal species were obtained from researchers of the Bio and Emerging Technology Institute (BETin), Addis Ababa, Ethiopia.

#### 4.5.3. Confirmation of Test Organism

Gram staining and biochemical identification were done to confirm the test organism. The test organisms were inoculated into MacConkey agar, Mannitol Salt agar, and Violet Red Glucose agar and were incubated at 35–37 °C for 24 h. After overnight incubation (35–37 °C), a gram reaction was carried out on the next day, followed by biochemical assays employing their biochemical properties. The isolated test organisms were kept on storage media at 2 to 8 °C until they were required. Each test organism was standardized using the 0.5 McFarland standard [63].

#### 4.5.4. Inoculum Preparation

The combination of 1% barium chloride dihydrate (BaCl_2_·2H_2_O) and 1% sulfuric acid (H_2_SO_4_) was used to prepare a 0.5 McFarland turbidity standard [64].

#### 4.5.5. Antimicrobial Test

The antimicrobial activity was predominantly evaluated qualitatively at testing concentrations of (100, 500, and 200 mg/mL) crude extracts using agar well diffusion techniques. The solution of the plant extracts was prepared by dissolving 200, 500, and 1000 mg/mL concentrations by resuspending the dried extracts with dimethyl sulfoxide (DMSO). A 10% *v*/*v* extract solution with sterile distilled water in a diluted dimethyl sulfoxide (10% *v*/*v* DMSO) was used as a diluent since it is a known universal solvent with no antibacterial activity at this concentration [65]. All the experiments were conducted in triplicates and the mean was recorded.

#### 4.5.6. Antimicrobial Agar Well Diffusion Assay

Mueller Hinton Agar (MHA) and potato dextrose agar were prepared according to the manufacturer’s instructions [64] and the medium was sterilized by autoclave at 121 °C for 15 min. A total of 20 mL of the sterile, molten, and 45 °C-cooled agar medium was put aseptically onto sterile, 90 mm-diameter Petri dishes. The dishes were then placed in the biological safety cabinet to harden at room temperature while maintaining sterility. The assay was carried out following the Clinical and Laboratory Standards Institute’s instructions (CLSI M100) guideline with slight modifications to meet the current experimental conditions [40]. After 16–18 h of incubation (37 °C and 25 °C for bacterial and fungal strains), organisms were sub-cultured in nutrient agar (bacteria) and Sabouraud Dextrose Agar (fungi) and in nutrient broth (bacteria) and SDB (for fungi) with adjusted 0.5 MacFarland standard as described above in the inoculum preparation, separately. 100 μL of predetermined test organisms were swabbed using sterile cotton swabs onto sterile Mueller Hinton agar plates and allowed to dry. By using blue tips, 8-mm diameter wells were punched into the inoculated agar media. Within 15 min, the wells were filled with a 100 μL 10% *v*/*v* extract solution. Likewise, the same volume of 10% *v*/*v* DMSO in distilled water and chloramphenicol (reference broad-spectrum antibiotic) was pipetted for negative and positive control, respectively. Before incubating, all dishes were preincubated at room temperature for 2 h to allow uniform diffusion of extract solution into the agar medium. Antimicrobial activity was assessed by measuring the diameter of the zone that inhibits bacterial growth in millimeters. Parallel tests for growth and stability quality controls were carried out and results were expressed as a mean of three replicates [64,65].

#### 4.5.7. Minimum Inhibition Concentration (MIC): Microdilution Method

On nutrient/brain heart infusion broth, the lowest concentration of a specific crude extract needed to inhibit the development of known test organisms in vitro was determined for each crude extract against the chosen test organisms. To do this, the crude extract was double-diluted to provide a spectrum of concentrations that were all twice as concentrated as previously. Making a stock solution was the first step, and sterilized nutrient/brain heart infusion broth was used to dilute each crude extract made from stock solutions [66].

#### 4.5.8. Preparation of Inoculum

Inoculum standardization is essential for precise and repeatable susceptibility testing. Overnight colonies from an agar medium were emulsified in a saline solution to prepare the inoculum. The recommended final inoculum size for broth dilution is 5 × 10^5^ CFU/mL [67].

#### 4.5.9. Colony Suspension Method

For this, 18 h- to 24 h-old cultures were taken from colonies by touching with a loop and transferring to saline solution or from broth suspension to saline solution and adjusted to be equivalent to a 0.5 McFarland standard. This was done in well-lit conditions by visually contrasting the appearance of black lines through the inoculum with McFarland standard suspensions (the inoculum and McFarland standard must be in the same-sized tubes). For the most frequently isolated species, it is estimated that a well-adjusted suspension contains 1.5 × 10^8^ CFU/mL. A dilution was made with a ratio of 10:990 of the bacterial suspension (1.5 × 10^8^ CFU/mL) with broth to get 1 × 10^6^ CFU/mL. Finally, by preparing the crude extract, when we add an equivalent amount of the bacterial suspension, we can get 1.5 × 10^5^ CFU/mL with a 1:2 ratio as the final inoculum [64,67].

#### 4.5.10. Viable Counts

To guarantee that the inoculum utilized for the test contains roughly 5 × 10^5^ CFU/mL, the viable count is crucial. The test was performed by diluting 10 μL from 1.5 × 10^8^ and diluting to 10 mL broth to get 5 × 10^5^ CFU/mL by calculation. Then we took 100 μL, spread it to nutrient agar, and incubated it for 24 h. Approximately 50 colonies are expected from an original inoculum of 5 × 10^5^ CFU/mL [67].

#### 4.5.11. Inoculation

Within 15 min of the inoculum preparation mentioned above, 1 mL of the adjusted inoculum was added to each tube containing 1 mL of the crude extract in the dilution series. The tubes were then mixed, and the inoculum was then incubated at 35 ± 2 °C for 16 to 20 h in an ambient air incubator. This results in a 1:2 dilution of both the inoculums and each antibacterial concentration.

#### 4.5.12. Minimum Bactericidal Concentration (MBC) Determination

A loopful of inoculum living test organisms from the MIC tubes was collected and streaked on new Mueller Hinton agar to find the lowest dose of a specific crude extract that can kill 99.9% of a specific bacterial strain that failed the MIC tests and did not show any detectable growth. After being incubated at 37 °C for 24 h, the streaked Mueller Hinton agar plates were checked for growth. A 99.9% bactericidal effect of the crude extract at that concentration, or MBC, is indicated by stained Mueller Hinton agar plates that do not display any growth [63].

### 4.6. Statistical Analysis

In this study, Microsoft Excel 2010 (Microsoft Corp., Redmond, WA, USA) was used for statistical analysis.

## 5. Conclusions

*D. angustifolia* flowers and leaves were found to be sources of phytochemicals such as extractable flavonoids and phenolic acids, and have considerable antibacterial and antifungal properties. From these activities observed and predicted from an online server, the flower can be considered as a therapeutic alternative mainly for its anti-aspergillosis activity and listeriosis to some extent. Following the phytochemical screening study managed previously, targeted HPTLC profiles of *D. angustifolia* leaves and flowers were obtained using a fairly validated method and found to be nearly the same for both extracts. Future work will emphasize isolating and characterizing active principles responsible for the aforementioned antimicrobial activities.

## Figures and Tables

**Table 1:** Comparison of DPPH radical scavenging activity, total phenol, and flavonoid content of DAL and DAF.

Sample	**DPPH Radical Scavenging Activity**		Total Phenolic Content (TPC) (mg/100 g)	Total Flavonoid Content (TFC) (mg/100 g)
	IC50	R^2^		
DAL	0.698 ± 0.002	0.995 ± 002	765.85 ± 16.95	700.66 ± 39.14
DAF	0.689 ± 0.005	0.997 ± 001	502.71 ± 7.56	488.23 ± 23
Ascorbic Acid	0.1237 ± 0.01	0.991 ± 0.02	-	-

DAL: *D. angustifolia* leaves; DAF: *D. angustifolia* flower; -: not applicable.

**Table 2:** Validation data for HPTLC analysis.

Parameters	Chlorogenic Acid	Gallic Acid	Kaempferol	Myricetin	Quercetin	Rutin	Syringic Acid
Wavelength	273 nm	273 nm	366 nm	366 nm	366 nm	366 nm	273 nm
Rf	0.35	0.62	0.49	0.63	0.48	0.075	0.70
R^2^	0.9977 ± 0.0006	0.9916 ± 0.0047	0.9965 ± 0.002	0.9862 ± 0.01	0.9845 ± 0.0048	0.9892 ± 0.0017	0.9912 ± 0.0044
Intercept	0.0008 ± 0	0.001 ± 0.0005	0.0018 ± 0.0011	0.0017 ± 0.0014	0.0012 ± 0.0005	0.0011 ± 0.0009	0.0025 ± 0.0009
Slope	0.0001 ± 0	0.0002 ± 0	0.0005 ± 0.0002	0.0006 ± 0.0003	0.0004 ± 0	0.0007 ± 0.0001	0.0003 ± 0.0001
LOD (µg/100 mL)	0.0540	0.0656	0.0264	0.0357	0.1317	0.0658	0.0720
LOQ (µg/100 mL)	0.1637	0.1987	0.0801	0.1083	0.3990	0.1992	0.2182
Range (ng/spot)	1600–100	1600–100	1600–100	1600–100	1600–100	1600–100	1600–100
Sensitivity	0.0001	0.0002	0.0005	0.0004	0.0003	0.0006	0.0003
Recovery (%)					98.91 ± 5.02	99.08 ± 4.92	
CV %	5.6341	10.69275	5.677567	11.96433	15.36685	8.75465	10.66925

Rf: retention factor; R^2^: regression coefficient; LOD: limit of detection; LOQ: limit of quantitation; CV %: percent of coefficient of variation.

**Table 3:** The contents (mg/100 g) of flavonoids and phenolic acids in the methanol extract of different parts of *D. angustifolia*.

Bioactive Compounds	DAL	DAF
Rutin	72.05 ± 0.47	63.09 ± 2.71
Quercetin	58.12 ± 0.62	51.38 ± 2.05
Kaempferol	ND	24.06 ± 0.81
Myrcetin	121.99 ± 0.76	98.78 ± 0.54
Chlorogenic acid	188.49 ± 1.78	165.62 ± 1.33
Gallic acid	32.26 ± 1.55	53.64 ± 1.21
Syringic acid	123.84 ± 1.1	176.85 ± 3.24

Data presented as mean SD (n = 3); ND: No data; DAL: *D. angustifolia* leaves; DAF: *D. angustifolia* flower.

**Table 4:** Probability of activity (Pa) summary for the flavonoids and phenolic acids.

S. No.	Activity	Standard Ascorbic Acid	Polyphenols						
			Quercetin	Rutin	Kaempferol	Myricetin	Gallic Acid	Gallic Acid	Chlorogenic Acid
1	Antibacterial	0.377	0.387	0.677	0.395	0.421	0.418	0.395	0.537
2	Antifungal	0.332	0.490	0.784	0.495	0.508	0.398	0.366	0.638

**Table 5:** The mean of inhibition zone of different crude extracts of assay of the antimicrobial test result.

No.	Test Microbial Organisms	200 mg/mL		500 mg/mL		1000 mg/mL	
		DAL	DAF	DAL	DAF	DAL	DAF
1	*Enterococcus faecalis* (ATCC79112)	9.0	8.0	NA	NA	NA	NA
2	*Escherichia coli* (ATCC25972)	0.0	0.0	0.0	0.0	0.0	0.0
3	*Klebsiella pneumonia* (ATTC70063)	0.0	0.0	0.0	0.0	0.0	0.0
4	*Listeria monocytogenes* (ATCC19115)	9.7	9.3	NA	NA	NA	NA
5	*Proteus mirabilis* (ATCC35659)	0.0	0.0	0.0	0.0	0.0	0.0
6	*Pseudomonas aeruginosa* (ATCC27853)	0.0	0.0	0.0	0.0	0.0	0.0
7	*Staphylococcus aureus* (ATTC25923)	0.0	0.0	0.0	0.0	0.0	0.0
8	*Aspergillus flavus*	0.0	0.0	16	24	NA	NA
9	*Fusarium fungal*	0.0	0.0	0.0	0.0	0.0	0.0

NA: not applicable.

**Table 6:** Results of minimum inhibition concentration (MIC) of microdilution.

No.	Test Microbial Organisms	5 μg/mL		10 μg/mL		20 μg/mL		40 μg/mL		80 μg/mL	
		L	F	L	F	L	F	L	F	L	F
1	*Enterococcus faecalis* (ATCC79112)	G	G	G	G	MIC	MIC	MBC	MBC	NA	NA
2	*Listeria monocytogenes* (ATCC19115)	G	G	G	G	MIC	MIC	MBC	MBC	NA	NA
3	*Aspergillus flavus*	G	G	G	G	G	G	MIC	MIC	MBC	MBC

L: leaf extract; F: flower extract; G: growth; MIC: minimum inhibition concentration; MBC: minimum bactericidal concentration; NA: not applicable.

## Data Availability

All generated data are enclosed within the manuscript and its supplementary file.

## Supplementary Materials

The following supporting information can be downloaded at https://www.mdpi.com/article/10.3390/molecules28062870/s1: Table S1: Phenolic acids peak at 273 nm for Flower and Leave samples; Table S2: Flavonoids peak at 366 nm for DAL and DAF samples.
