# Supplementary material for: Targeted HPTLC Profile, Quantification of Flavonoids and Phenolic Acids, and Antimicrobial Activity of Dodonaea angustifolia (L.f.) Leaves and Flowers
Source: Molecules. 2023 Mar 22;28(6):2870. doi: 10.3390/molecules28062870 (PMC10052987; doi:10.3390/molecules28062870)
Supplement: Supplementary file 1 [file molecules-28-02870-s001.zip › molecules-2285248-supplementary.pdf]

Table S1. Phenolic acids peak at 273 nm for Flower and Leave samples.

Sample	Peak Number	Max Rf	Height	Height %	Area	Area %	Assigned substance
DAF	1	0.017532	0.074595	7.18096	0.001192	1.750044	
DAF	2	0.227922	0.058172	5.599932	0.001772	2.601398	
DAF	3	0.334416	0.078618	7.568258	0.003303	4.84773	Chlorogenic acid
DAF	4	0.374675	0.090487	8.71078	0.003507	5.14676	
DAF	5	0.470779	0.026823	2.582139	0.001011	1.484161	
DAF	6	0.573377	0.026614	2.562002	0.000723	1.061726	
DAF	7	0.672078	0.05029	4.841246	0.001057	1.55081	Gallic acid
DAF	8	0.738312	0.135771	13.07006	0.003544	5.201121	Syringic acid
DAF	9	0.814935	0.497422	47.88463	0.052026	76.35625	
DAL	1	0.134416	0.027493	2.143005	0.000537	0.533998	
DAL	2	0.230519	0.043594	3.397995	0.001477	1.4686	
DAL	3	0.37987	0.12896	10.05202	0.005599	5.567087	Chlorogenic acid
DAL	4	0.475974	0.067411	5.25449	0.003222	3.20352	
DAL	5	0.577273	0.073522	5.730762	0.003664	3.643362	Gallic acid
DAL	6	0.674675	0.094064	7.332004	0.002549	2.534259	Syringic acid
DAL	7	0.821429	0.657575	51.25578	0.07386	73.43587	
DAL	8	0.930519	0.190309	14.83395	0.009669	9.613304	

Table S2. Flavonoids peak at 366 nm for DAL and DAF samples.

Sample	Group of std	Peak Number	Max Rf	Height	Height %	Area	Area %	Assigned substance
DAF	R+Q	1	0.029221	0.072513	3.550314	0.00104	1.463886	
DAF		2	0.056494	0.140997	6.903387	0.002376	3.343433	
DAF		3	0.082468	0.165065	8.08176	0.002313	3.255528	Rutin 3
DAF		4	0.094156	0.271733	13.30437	0.00567	7.979827	
DAF		5	0.159091	0.02071	1.013992	0.0004	0.562942	
DAF		6	0.312338	0.043578	2.133647	0.001275	1.79383	
DAF		7	0.462987	0.065191	3.191805	0.002315	3.258083	
DAF		8	0.485065	0.126123	6.175144	0.001934	2.721258	Quercetin 3
DAF		9	0.501948	0.213575	10.45688	0.005397	7.595727	
DAF		10	0.548701	0.395632	19.37059	0.016738	23.55496	
DAF		11	0.614935	0.527319	25.81811	0.0316	44.47053	
DAF	M+K	1	0.052597	0.047943	1.541725	0.000951	0.720274	
DAF		2	0.078571	0.036902	1.186688	0.00042	0.318369	
DAF		3	0.126623	0.155506	5.000696	0.00449	3.401952	
DAF		4	0.165584	0.186874	6.009414	0.006745	5.111274	

DAF		5	0.472078	0.034674	1.115025	0.001648	1.248498	Kaempferol
DAF		6	0.578571	0.067373	2.166574	0.002073	1.570525	
DAF		7	0.625325	0.24921	8.014023	0.006778	5.136223	Myrcetin
DAF		8	0.648701	0.42339	13.61522	0.01393	10.555	
DAF		9	0.696753	0.51126	16.44093	0.028483	21.58292	
DAL	Q+R	1	0.025325	0.024707	1.039441	0.000236	0.268393	
DAL		2	0.069481	0.156982	6.604309	0.003269	3.718966	Rutin 3
DAL		3	0.096753	0.284615	11.97392	0.007163	8.149491	
DAL		4	0.168182	0.035254	1.483175	0.000838	0.953951	
DAL		5	0.188961	0.031149	1.310454	0.000428	0.486482	
DAL		6	0.275974	0.025409	1.068975	0.000698	0.794222	
DAL		7	0.321429	0.051414	2.163017	0.001583	1.801545	
DAL		8	0.352597	0.036073	1.517616	0.000761	0.865601	
DAL		9	0.466883	0.108408	4.560785	0.003494	3.974727	
DAL		10	0.488961	0.176523	7.426442	0.002646	3.010885	Quercetin 3
DAL		11	0.505844	0.307415	12.93313	0.008269	9.407533	
DAL		12	0.547403	0.338216	14.22895	0.010977	12.4885	
DAL		13	0.617532	0.591673	24.89202	0.036178	41.16094	
DAL		14	0.785065	0.209119	8.797761	0.011355	12.91877	
DAL	M+K	1	0.026623	0.045109	0.933607	0.000431	0.21226	
DAL		2	0.053896	0.020298	0.420102	0.00036	0.17732	
DAL		3	0.082468	0.025704	0.531995	0.000351	0.172856	
DAL		4	0.129221	0.166439	3.444747	0.00442	2.175436	
DAL		5	0.159091	0.268935	5.566098	0.009171	4.513845	
DAL		6	0.265584	0.046146	0.955079	0.002198	1.081581	
DAL		7	0.408442	0.046574	0.963942	0.001984	0.976351	
DAL		8	0.491558	0.086044	1.780837	0.002453	1.207428	
DAL		9	0.555195	0.099654	2.06252	0.002262	1.113144	
DAL		10	0.62013	0.395708	8.189892	0.019586	9.639872	
DAL		11	0.644805	0.438288	9.071162	0.012453	6.129081	Myrcetin
DAL		12	0.695455	0.649049	13.43325	0.038988	19.189	
DAL		13	0.855195	0.245164	5.074119	0.013346	6.568701	
